# Unusual Etiology of Acute Wrist Pain: Acute Calcific Tendonitis of the Flexor Carpi Ulnaris Mimicking an Infection

**DOI:** 10.1155/2018/2520548

**Published:** 2018-11-14

**Authors:** Man R. Shim

**Affiliations:** ^1^Department of Medicine, Rheumatology Division, David Geffen School of Medicine, University of California, Los Angeles, Los Angeles, CA, USA; ^2^UCLA Health Beverly Hills, 8641 Wilshire Blvd., Suite 210, Beverly Hills, CA 90211, USA

## Abstract

Acute calcific tendonitis is a common cause of musculoskeletal pain. However, it seldom affects the hand and wrist. For that reason, it is frequently mistaken for more common etiologies. This study reports a case of acute calcific tendinitis of the flexor carpi ulnaris, which was initially misdiagnosed as cellulitis, in a 65-year-old woman, who was unnecessarily prescribed with antibiotics. However, further evaluation confirmed the correct diagnosis of acute calcific tendinitis and her symptoms were subsequently resolved within 2 weeks with rest, wrist immobilization, and an intake of anti-inflammatories. This case underscores the need for the physicians to be aware of this less common but important cause of acute wrist pain in order to prevent misdiagnosis and avoid unnecessary medical treatments.

## 1. Introduction

Acute calcific tendonitis (ACT) is an acute inflammatory condition of unknown etiology caused by the pathologic deposition of calcium hydroxyapatite crystals in tendons. While the shoulder and hip are the most commonly affected sites, ACT is an uncommon inflammatory disorder of the hand and wrist [1]. Although calcium deposition around the shoulder may be asymptomatic at times, the presence of calcium deposits in the hand and wrist is usually manifested by severe pain, swelling, and erythema [2]. The calcium deposition of the flexor carpi ulnaris is the most common form of ACT that affects the hand and wrist [3]. However, due to its rare incidence, ACT of the hand and wrist is often mistaken for other conditions such as gout, pseudogout, fracture, or infection [4]. As such, it is not uncommon for the affected patients to undergo unnecessary medical treatments and invasive procedures including surgery [5]. Because the natural history is of a self-limiting condition and plain radiographs usually confirm the diagnosis [2–4], the clinicians need to become familiar with this entity in order to minimize avoidable errors in diagnosis and treatment as illustrated in this report.

## 2. Case Report

A 65-year-old, left hand-dominant female with no significant past medical history presented to her primary care physician with acute onset of progressive left wrist pain, erythema, and swelling of a five-day duration. The patient denied any history of recent trauma. She was diagnosed with cellulitis and was given ceftriaxone 1000 mg intramuscular injection in the clinic followed by cephalexin 500 mg to take orally four times a day as an outpatient. However, her aforementioned symptoms did not improve with prescribed antimicrobial therapy. As such, she was subsequently referred to the rheumatology department two days later for further evaluation and management.

**Figure 1:**
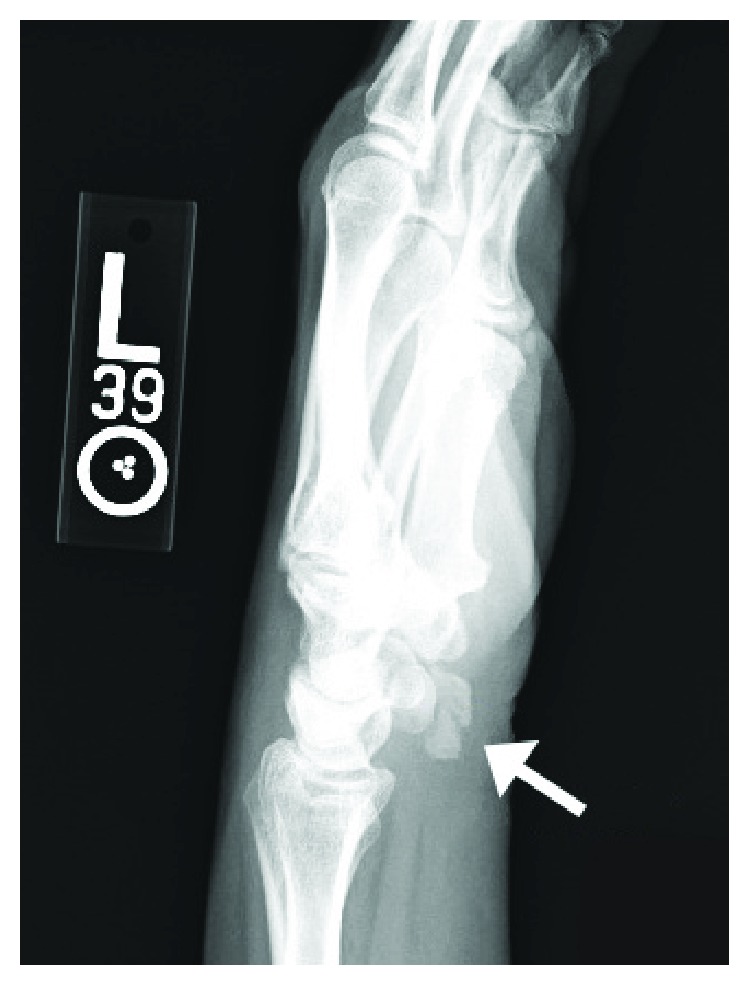
Oblique view of the wrist joint revealing acute calcific tendinitis of the flexor carpi ulnaris with a 1.3 × 0.7 cm calcium deposit (arrow) anterior to the pisiform.

The patient was afebrile with stable vital signs. Her white blood cell count and inflammatory markers were also within the normal limits. On physical examination, the patient had tenderness, edema, erythema, and warmth over the ulnar aspect of the left volar wrist. The pain was aggravated by ulnar deviation and flexion of the wrist joint. Plain radiographs of the left wrist revealed a 1.3 × 0.7 cm area of calcific deposit about the volar aspect of the pisiform bone (Figure 1). Upon further questioning, specifically about repetitive activities, she endorsed typing on the keyboard all day at work and having cleaned horse stalls over the weekend prior to the onset of her symptoms.

Although her clinical presentation was initially concerning for an infectious etiology, taking a thorough history, performing a comprehensive physical examination and a careful review of the radiographs confirmed the diagnosis of acute calcific tendonitis of the flexor carpi ulnaris. As such, her ongoing antibiotic treatment was discontinued. Instead, she was prescribed nonsteroidal anti-inflammatory drugs (NSAIDs) and a wrist splint for immobilization. Her symptoms subsequently improved significantly within 48 hours and she was symptom-free at 2-week follow-up visit.

## 3. Discussion

ACT of the hand and wrist was first described by Cohen in 1924 [6]. Since that time, there have been several reports describing its occurrence sporadically in the literature. Its etiology is currently not well known. Some of the hypotheses include microvascular trauma and local tissue hypoxia leading to pathologic deposition of calcium, which results in inflammatory responses including tenderness, swelling, erythema, and restriction of motion secondary to pain at the affected site [2, 7]. Although major antecedent trauma is rare, a history of minor trauma or stress injury has been reported in up to one-third of patients [7]. However, this theory conflicts with 12 cases of calcific tendonitis of the hand and wrist reported by Moyer et al., where initiating trauma or repetitive strain could not be readily identified [3]. Regardless, the symptoms experienced by the patients with ACT of the hand and wrist are postulated to be due to resorptive phase or rupture of a calcific deposit into the adjacent soft tissue and not as a direct result of the calcification itself [7].

Due to its rare occurrence and overlap of its clinical symptoms with other entities, ACT of the hand and wrist is frequently misdiagnosed as acute infection, fracture, tenosynovitis, or crystalline arthropathy [4]. Many of the cases reported in the literature were often inappropriately treated with antibiotics and even underwent unnecessary surgery [6]. As such, taking a careful clinical history, performing a comprehensive physical examination, and ordering appropriate blood work and radiographic studies are of paramount importance. Basic laboratory and microbiological tests are usually normal in ACT [8]. Radiographically, fluffy and amorphous calcifications are typically found at the affected tendons, which often allow the correct diagnosis to be made. Additional oblique radiographs may be required because small calcifications can be easily missed with only a posterior-anterior view. Rarely, more advanced imaging studies such as ultrasound, computed tomography, and magnetic resonance imaging (MRI) are needed to confirm or exclude the diagnosis of ACT. On MRI, calcifications appear as focal areas of low signal on all pulse sequences, typically located at or near the tendon insertion [9].

ACT of the hand and wrist is usually a self-limiting process, and treatment is conservative. Traditional therapies include rest, oral NSAIDs, and splint for immobilization. Within several weeks, the symptoms of ACT usually improve significantly or resolve completely, and calcific deposits on radiographs also typically disappear or markedly decrease in size [2–4, 7]. More invasive procedures including local anesthetic or steroid injections, puncture for aspirating the calcium deposit, and surgical evacuation are no longer advocated and usually are reserved for severe cases which conservative measures have failed and symptoms last for more than several weeks [10].

## 4. Key Points

- This report illustrates a usual presentation of ACT of the hand and wrist, which was initially misdiagnosed as cellulitis and treated unnecessarily with antibiotics
- ACT of the hand and wrist is often unrecognized because of its rarity, nonspecific clinical presentation which can mimic other conditions, and lack of familiarity amongst the practicing physicians
- Because this condition is usually self-limiting and characteristic radiological findings typically lead to an accurate diagnosis, an increased awareness of this condition is much needed in order to prevent misdiagnosis and avoid unnecessary treatments
